# Development of Resorbable Phosphate-Based Glass Microspheres as MRI Contrast Media Agents

**DOI:** 10.3390/molecules29184296

**Published:** 2024-09-10

**Authors:** Jesús Molinar-Díaz, Andi Arjuna, Nichola Abrehart, Alison McLellan, Roy Harris, Md Towhidul Islam, Ahlam Alzaidi, Chris R. Bradley, Charlotte Gidman, Malcolm J. W. Prior, Jeremy Titman, Nicholas P. Blockley, Peter Harvey, Luca Marciani, Ifty Ahmed

**Affiliations:** 1 Advanced Materials Research Group, Faculty of Engineering, University of Nottingham, Nottingham NG7 2RD, UK; jesus.molinardiaz3@nottingham.ac.uk (J.M.-D.); andi.arjuna@nottingham.ac.uk (A.A.); towhid.islam@nottingham.ac.uk (M.T.I.); 2 Composites Research Group, Faculty of Engineering, University of Nottingham, Nottingham NG7 2GX, UK; 3 Faculty of Pharmacy, Hasanuddin University, Makassar 90245, Indonesia; 4 Nottingham Digestive Diseases Centre, Translational Medical Sciences, School of Medicine, University of Nottingham, Nottingham NG7 2UH, UK; nichola.abrehart@nottingham.ac.uk (N.A.); luca.marciani@nottingham.ac.uk (L.M.); 5 School of Chemistry, University of Nottingham, Nottingham NG7 2RD, UK; alisondalymclellan@live.com (A.M.); charlotte.gidman1@nottingham.ac.uk (C.G.); jeremy.titman@nottingham.ac.uk (J.T.); peter.harvey@nottingham.ac.uk (P.H.); 6 Research Design Service East Midlands, Queen’s Medical Centre, Nottingham NG7 2UH, UK; roy.harris1@nottingham.ac.uk; 7 School of Life Sciences, University of Nottingham Medical School, Queen’s Medical Centre, Nottingham NG7 2UH, UK; lpxaaa@nottingham.ac.uk (A.A.); nicholas.blockley@nottingham.ac.uk (N.P.B.); 8 Sir Peter Mansfield Imaging Centre, School of Medicine, University of Nottingham, Nottingham NG7 2QX, UK; christopher.bradley@nottingham.ac.uk (C.R.B.); malcolm.prior@nottingham.ac.uk (M.J.W.P.)

**Keywords:** Magnetic Resonance Imaging, phosphate-based glasses, oral contrast agents, porous microspheres, resorbable materials

## Abstract

In this research, resorbable phosphate-based glass (PBG) compositions were developed using varying modifier oxides including iron (Fe_2_O_3_), copper (CuO), and manganese (MnO_2_), and then processed via a rapid single-stage flame spheroidisation process to manufacture dense (i.e., solid) and highly porous microspheres. Solid (63–200 µm) and porous (100–200 µm) microspheres were produced and characterised via SEM, XRD, and EDX to investigate their surface topography, structural properties, and elemental distribution. Complementary NMR investigations revealed the formation of Q^2^, Q^1^, and Q^0^ phosphate species within the porous and solid microspheres, and degradation studies performed to evaluate mass loss, particle size, and pH changes over 28 days showed no significant differences among the microspheres (63–71 µm) investigated. The microspheres produced were then investigated using clinical (1.5 T) and preclinical (7 T) MRI systems to determine the *R*_1_ and *R*_2_ relaxation rates. Among the compositions investigated, manganese-based porous and solid microspheres revealed enhanced levels of *R*_2_ (9.7–10.5 s^−1^ for 1.5 T; 17.1–18.9 s^−1^ for 7 T) and *R*_1_ (3.4–3.9 s^−1^ for 1.5 T; 2.2–2.3 s^−1^ for 7 T) when compared to the copper and iron-based microsphere samples. This was suggested to be due to paramagnetic ions present in the Mn-based microspheres. It is also suggested that the porosity in the resorbable PBG porous microspheres could be further explored for loading with drugs or other biologics. This would further advance these materials as MRI theranostic agents and generate new opportunities for MRI contrast-enhancement oral-delivery applications.

## 1. Introduction

Magnetic resonance imaging (MRI) is a safe medical imaging technique that employs a strong magnetic field and radio waves to transmit and receive radio signals from water molecules in various tissues in the body [1]. Spatial and physicochemical information about the tissues is encoded in the received signal, allowing three-dimensional images of the body to be reconstructed and subsequently used to diagnose disease or monitor patient-specific treatments [2]. The most common magnetic resonance (MR) images used clinically are based on either *T*_1_-weighted (*T*_1_w) or *T*_2_-weighted (*T*_2_w) sequences. These sequences are named after the two main relaxation time constants, *T*_1_ and *T*_2_, with which the tissue signal goes back to its baseline state after radiofrequency excitation [3]. Longitudinal (*R*_1_ = 1/*T*_1_) and transverse (*R*_2_ = 1/*T*_2_) relaxation rates of water protons are dependent on the physicochemical state of different tissue and fluid components in the body, which drive the richness of contrast in the MR images and can enable differentiation between healthy and diseased tissue [4]. The key concept and the main reason MRI demand has grown exponentially in diagnostic radiology is that the MRI scanner operator can control the specific contributions of *R*_1_ and *R*_2_ in the MR images, and therefore can manipulate the range of contrast and brightness to highlight specific tissues and body structures as desired. When endogenous contrast cannot be exploited sufficiently, the contrast and brightness manipulation of MR images can be enhanced using contrast agents (CAs), which are substances with the ability to strongly impact local *R*_1_ and/or *R*_2_ values [2]. 

Intravenous contrast agents, such as gadolinium chelates [5], have routinely been used in MRI clinical scan examinations, although concerns over their safety have recently emerged [6]. For example, safety reports on the use of gadolinium-based CAs have shown accumulation in the brain, heart, and blood vessels, and have also been reported to cause severe issues for patients with kidney failure and renal insufficiency, leading to product withdrawals [7,8]. Iron oxide contrast agents, such as magnetic core/polymer shell microspheres, have also been used in MRI applications (i.e., Dynabeads^®^) [9]. In the mid-1990s, several iron-based MRI CAs were developed for imaging the liver and other cells of the reticuloendothelial system (RES). These CAs were marketed under the names of Feridex^®^ (Endorem^®^), Resovist^®^ (Cliavist^®^), and Sinarem^®^ (Combidex^®^) [10,11,12,13,14,15]. These CAs were colloid suspensions consisting of very small (30–200 nm) clusters of iron-containing molecules forming single magnetic domains. In *T*_2_-weighted images, Ferridex^®^ (administered as a thick saline solution via intravenous infusion over 30 min) showed a normal-functioning liver as dark, while metastatic lesions remained bright [7]. Ultimately, these CAs failed due to their diagnostic utility not being effective enough, and by 2009 manufacturers had ceased commercial production of all RES agents [15,16], leaving only Resovist^®^ available for liver imaging in a few countries [17] and Feraheme^®^ for iron deficiency anaemia and MRI (off-label) [18,19]. 

Oral contrast agents for MRI have been used to distend the lumen of the gastrointestinal (GI) tract while providing bright or dark contrast between the lumen and the bowel walls in different MRI sequences. This approach can allow a clear assessment of the bowel wall’s thickness or the presence of polyps without exposing the patient to harmful ionising radiation from X-rays. Gadolinium-based MRI contrast agents, such as the positive contrast enteral formulation Gd-DTPA (Magnevist^®^), and manganese-based (LumenHance^®^), have also long been discontinued [2]. Specifically, LumenHance^®^ was withdrawn because of poor sales and because high doses of free manganese ions can cause a neurodegenerative disorder called manganism, which resembles Parkinson’s disease symptoms [19]. As of 2021, no Mn-based contrast agent was commercially available [20]. Other commercial products, such as oral suspensions of siloxane-coated magnetite nanoparticles (GastroMARK^®^/Lumirem^®^), a sugar flavoured alcohol-based beverage (Breeza), a polyethylene glycol preparation (MiraLAX), or low-concentration barium sulphate (VoLumen), have also been used [21]. However, commonly reported problems with these agents include palatability, difficulty ingesting the doses required to achieve sufficient bowel distension, abdominal pain, and diarrhoea, especially in younger patients [22]. 

A variety of beverages have also previously been explored for MR contrast, especially for use with children. These include pineapple and blueberry juice, due to their relatively high concentration of manganese [23]. Naturally occurring manganese has also previously been shown to be a safe contrast agent [24]. Although safe and palatable [25], these juices are absorbed too quickly by the small intestine due to their high water content, and they can lose the ability to distend the distal GI tract [26]. 

At present, there are no oral contrast agents (OCAs) dedicated for MRI use that enable imaging of the entire GI tract (i.e., from oesophagus to large intestine) [27]. Alternatively, other imaging modalities have developed OCAs for the GI tract. For example, current procedures to diagnose some upper GI conditions have used X-rays alongside barium-based drinks that fill and distend the lumen of the GI tract and provide good contrast not otherwise available on X-ray images. However, X-ray imaging also provides an ionising radiation dose to the patients [28], which can increase the overall risk of cancer, making barium X-rays, and especially dynamic fluoroscopic examinations, particularly unsuitable for children and women of childbearing age. With MRI capabilities and applications rapidly expanding in hepatology and the GI area, the need for relevant contrast agents is more apparent than ever.

In this study, we developed and characterised fully resorbable phosphate-based glass (PBG) dense (i.e., bulk, solid) and highly porous microspheres and investigated their effect on MRI relaxation rates (*R*_1_ and *R*_2_). PBG microspheres have been explored for several potential applications in healthcare, e.g., bone tissue regeneration [29] and radiotherapy [30]. In particular, porous microspheres provide additional benefits when compared to dense counterparts, as the pore cavities enable payloads (i.e., cells, other biologics, drugs etc.) to be incorporated, making them suitable candidates for drug delivery applications. 

In this context, phosphate-based glasses doped with varying oxides including iron (Fe^3+^), copper (Cu^2+^), and manganese (Mn^4+^) oxides were explored in this study. These formulations were developed and processed into microspheres via our facile, rapid, single-stage flame spheroidisation process. Furthermore, characterisation was performed to confirm their morphology and chemistry using scanning electron microscopy (SEM) and energy dispersive x-ray spectroscopy (EDX). Structural investigations were also performed using X-ray diffraction (XRD) to confirm the nature of the materials produced, and degradation studies were carried out to determine their resorption timeframe. Complementary structural analysis was conducted via nuclear magnetic resonance (NMR) studies. These microspheres were suspended in a model hydroxyethyl cellulose water/sodium chloride solution for measurement of *R*_1_ and *R*_2_ relaxation rates in vitro using a 1.5 T and a 7 T MRI scanner.

## 2. Results

### 2.1. Microsphere Morphologies 

**Figure 1:**
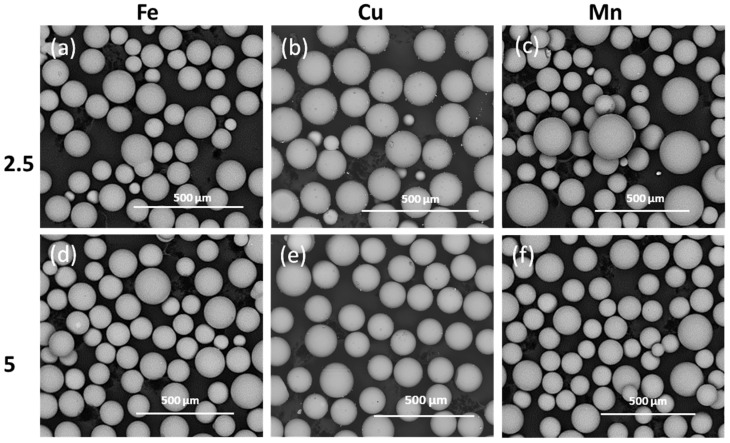
SEM images of flame spheroidised-processed solid microspheres (SMS) P45 (63–200 µm) with modified compositions: Fe–(**a**) 2.5%, (**d**) 5%; Cu–(**b**) 2.5%, (**e**) 5%; and Mn–(**c**) 2.5%, (**f**) 5%.

Figure 1a–f shows low magnification electron microscopy images of flame spheroidised solid (i.e., bulk, dense) microspheres (SMS) P45 (63–200 µm), with modified Fe, Cu, and Mn concentrations. SEM investigations demonstrated that SMS were produced (and were then sieved to obtain particle size range between 63–200 µm). After SEM imaging, the SMS products were further sieved to a very narrow size distribution range (63–71 µm) for degradation studies, and MRI relaxation measurements used particle sizes within the 100–200 µm size ranges.

**Figure 2:**
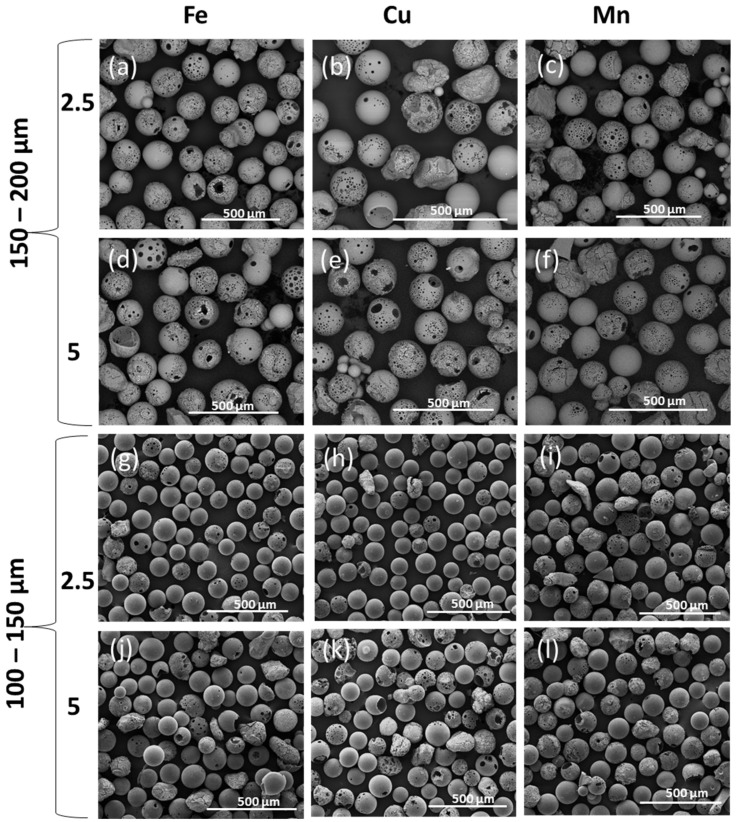
SEM images of flame spheroidised-processed porous microspheres (PMS) P45, in the ranges of (**a**–**f**) 150–200 µm and (**g**–**l**) 100–150 µm, with modified compositions: Fe–(**a**,**g**) 2.5%, (**d**,**j**) 5%; Cu–(**b**,**h**) 2.5%, (**e**,**k**) 5%; and Mn–(**c**,**i**) 2.5%, (**f**,**l**) 5%.

Figure 2a–l show low magnification SEM images of flame spheroidised porous glass microspheres (PMS) P45 samples sieved in two size ranges (100–150 µm, and 150–200 µm). These were modified with Fe, Cu, and Mn concentrations. Notably, porous microspheres produced in the range of 150–200 µm, generated a consistent yield (see Figure 2a–f) with only a few solid and/or irregular shaped particles observed. For smaller size range distributions, PMS (100–150 µm), a mixture of both solid and porous microspheres along with some irregular shaped particles was observed (Figure 2g–l). Overall, better porosity quality was observed for the larger of the two size ranges, as seen in Figure 2a–f.

### 2.2. Structural Characterisation

**Figure 3:**
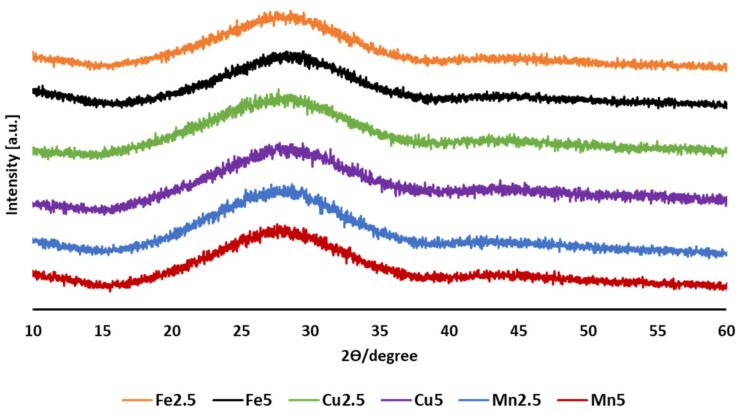
XRD patterns for flame spheroidised-processed solid glass microspheres (SMS) P45 with modified Fe, Cu, and Mn compositions.

Figure 3 presents XRD profiles for the SMS microspheres from all the formulations produced. The absence of any sharp distinct crystalline peaks suggested that the materials produced via flame spheroidisation remained in an amorphous state.

**Figure 4:**
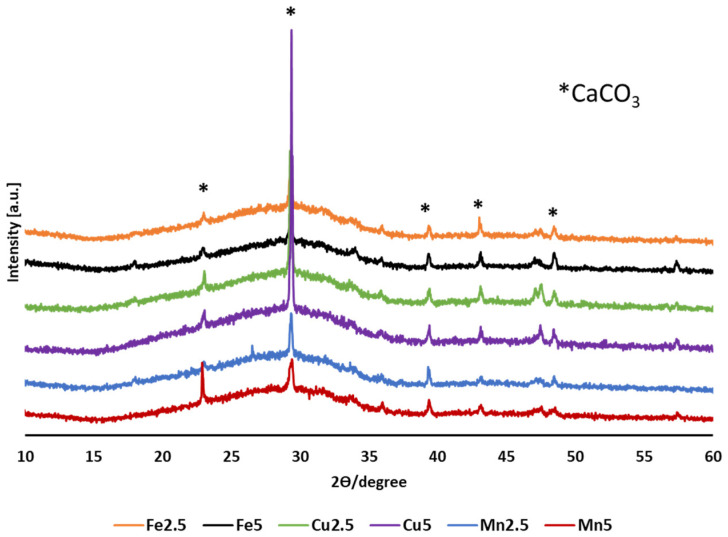
XRD patterns for flame spheroidised-processed porous microspheres (PMS) P45 with modified Fe, Cu, and Mn compositions.

Figure 4 shows the XRD profiles for the PMS produced, revealing a combination of an amorphous hump along with distinct crystalline peaks which were matched to CaCO_3_ (PDF 01-071-3699) for all cases. These CaCO_3_ signatures were attributed to unreacted CaCO_3_-based porogen, either from remaining unreacted porogen or as porogen remnants on or within the porous microspheres produced. 

### 2.3. Compositional Analysis 

Molar concentrations (mol%) corresponding to the solid and porous microspheres developed are summarised in Table 1. It was evident that P_2_O_5_ and CaO concentration levels were similar among all SMS and PMS samples, except for SMS P45- Cu2.5 and Cu5, which showed lower levels of P_2_O_5_ and higher levels of CaO. In particular, Na_2_O showed higher concentrations for PMS (12–21 wt%) when compared to SMS (7–13 wt%). This effect was most likely influenced by the incorporation of Na_2_CO_3_ porogen, which was used for porous microsphere formation. Furthermore, it was confirmed that Fe_2_O_3_, CuO, and MnO_2_ concentrations were in good agreement with the expected glass formulations (Table 4).

**Figure 5:**
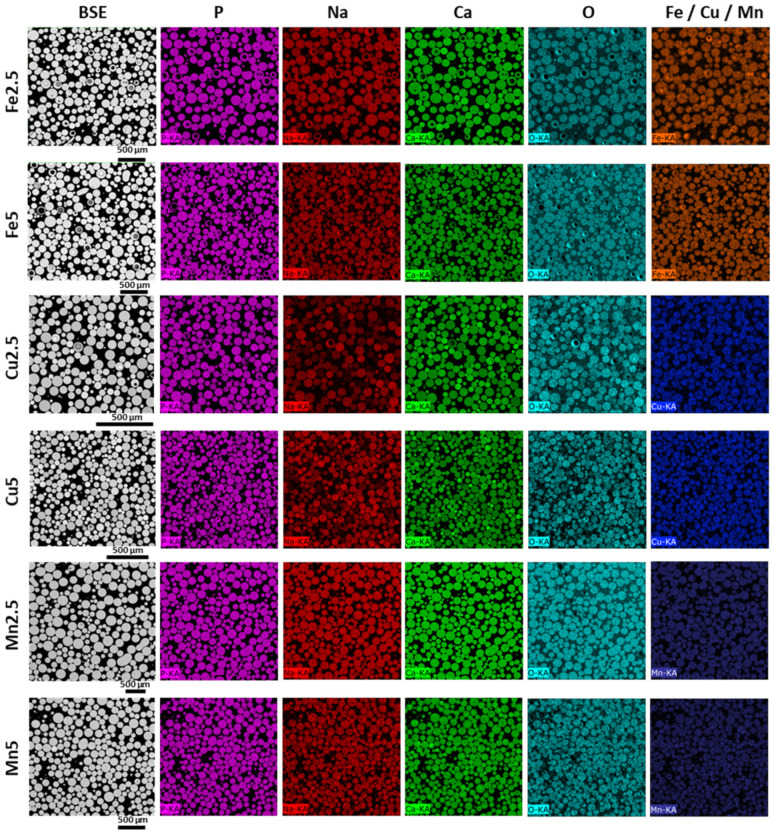
BSE images and EDX elemental mapping of flame spheroidised-processed solid glass microspheres (SMS) P45, following sectioning, illustrating modified Fe, Cu, and Mn compositions. (The colours represent: purple for phosphate, red for sodium, green for calcium, light blue for oxygen, blue for copper, and dark blue for manganese).

EDX mappings provided additional information related to elemental distribution within the samples. Importantly, it was observed for the PMS that irregularly shaped particles (very few) showed rich levels of calcium, which were attributed to excess (unreacted) CaCO_3_ porogen. Nonetheless, the microsphere products showed high levels of compositional uniformity for all cases (SMS and PMS). Figure 5 presents correlated BSE images and EDX elemental mappings of resin-embedded and sectioned SMS samples corresponding to the sample sets shown in Figure 1, confirming well-defined solid morphologies of flame spheroidised-products. EDX mapping revealed good levels of homogeneous compositional distribution among SMS formulations.

**Figure 6:**
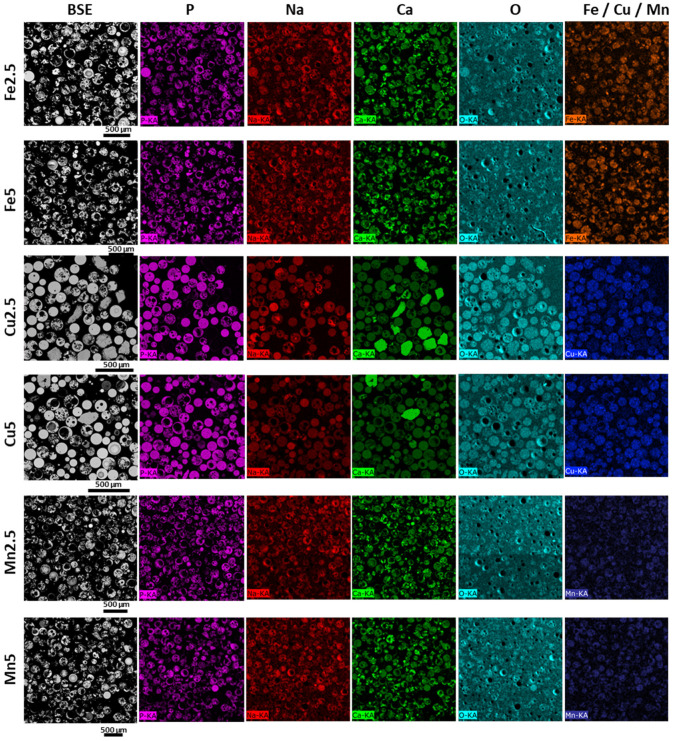
BSE images and EDX elemental mapping of flame spheroidised-processed porous glass microspheres (PMS) P45 (100–200 µm), following sectioning, illustrating the modified Fe, Cu, and Mn compositions and revealing good levels of interconnected porosity among the samples. (The colours represent: purple for phosphate, red for sodium, green for calcium, light blue for oxygen, blue for copper, and dark blue for manganese).

Figure 6 shows correlated BSE images and EDX elemental mappings of resin-embedded and sectioned PMS samples, extracted from sample sets imaged in Figure 2, revealing fine-scale morphological details of flame spheroidised-products. A variety of porous morphologies and high levels of interconnected porosity for PMS formulations was evident.

Nuclear magnetic resonance (NMR) analysis was used to assess the concentration of different Q^n^ phosphate species within the flame spheroidised SMS and PMS samples produced. Figures S1–S3 (ESI) show ^31^P NMR spectra for Fe, Cu, and Mn, respectively. As summarised in Table 2, for the Fe and Cu based SMS with an increase in Fe and Cu concentrations from 2.5 mol% to 5 mol%, the phosphate species observed within the glass were predominantly Q^2^ and Q^1^ meta- and pyrophosphates. The two peaks consistently observed were in the ranges of −23.9 to −20.9 ppm and −8.1 to −7.9 ppm. These peaks were associated with the Q^2^ and Q^1^ species, respectively. The slight shifts observed were attributed to the addition of Fe_2_O_3_ and CuO to the P45 glass formulation. Conversely, for the case of Mn solid microspheres, it was noted that as the Mn concentration increased from 2.5 mol% to 5 mol%, only Q^1^ phosphate species were observed. The absence of Q^0^ in Mn5 was attributed to the incorporation of MnO_2_ into the P45 glass. Interestingly, the NMR spectra for both Mn2.5 and Mn5 SMS revealed a dominant peak representing Q^1^ (−22 ppm). The NMR Q^n^ species observed for the solid microspheres were equivalent to the starting glass formulation Q^n^ values (i.e., prior to flame spheroidisation).

For the case of Fe, Cu, and Mn-based PMS, the phosphate species observed within the microspheres were mainly Q^2^ (−23.1 to −20.8 ppm), Q^1^ (−8 to −5.3 ppm), and Q^0^ (2.5 to 3.9 ppm) meta-, pyro- and orthophosphates, respectively. It was suggested that such distributions among the Q^n^ phosphate species may be indicative of a change in composition due to processing effects, such as the incorporation of porogen remnants into the P45 glass.

### 2.4. Degradation Studies

**Figure 7:**
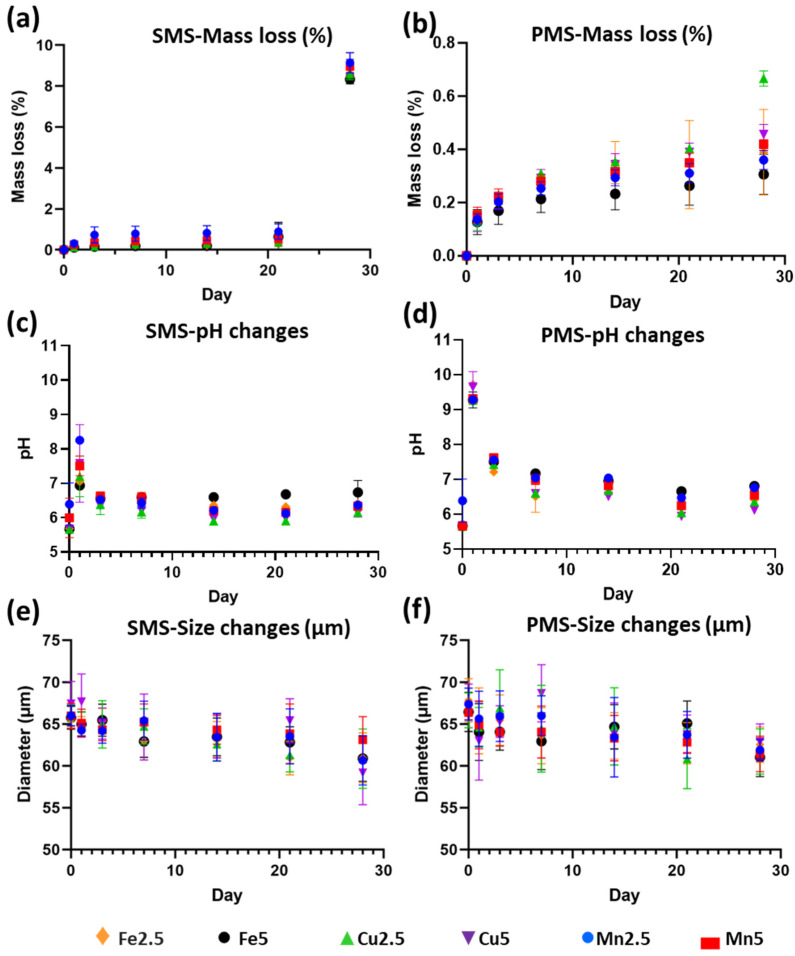
Degradation studies on: (**a**,**b**) Mass loss (%), (**c**,**d**) pH change, and (**e**,**f**) microsphere size (µm), as a function of immersion time (day 1, 3, 7, 14, 21, and 28) for (**a**,**c**,**e**) solid, and (**b**,**d**,**f**) porous microspheres of Fe2.5, Fe5, Cu2.5, Cu5, Mn2.5, and Mn5 over 28 days. SMS (solid microspheres); PMS (porous microspheres).

The degradation profiles of the microspheres immersed in milli-Q water are shown in Figure 7a slight decrease in mass for all SMS glass formulations (Figure 7a) was observed by day 21, with an evident mass decrease (~8%) on day 28. For the PMS (Figure 7b), a slight and gradual decrease in mass (~1%) occurred from day 1 to day 28. 

The pH changes for the milli-Q water medium with SMS (Figure 7c) and PMS (Figure 7d) increased at day-1 for all compositions. The elevated pH (~9–10) at day 1 for PMS formulations, as compared to SMS lower pH (~6.5–8.5), was attributed to the presence of CaCO_3_. After day-3, the pH for all glass formulations in both SMS and PMS, reached values in the range of ~6–6.5, and remained constant until day 28. 

Based on the initial outcomes above, a follow-up study was carried out using a distinct close particle size range against immersion time for SMS (Figure 7e) and PMS (Figure 7f). This study revealed decreasing profiles over time, with statistically insignificant (*p* > 0.05) differences between the formulations tested. However, a larger variation in the data set was seen for the PMS.

### 2.5. Measurement of MR Relaxation Times

**Figure 8:**
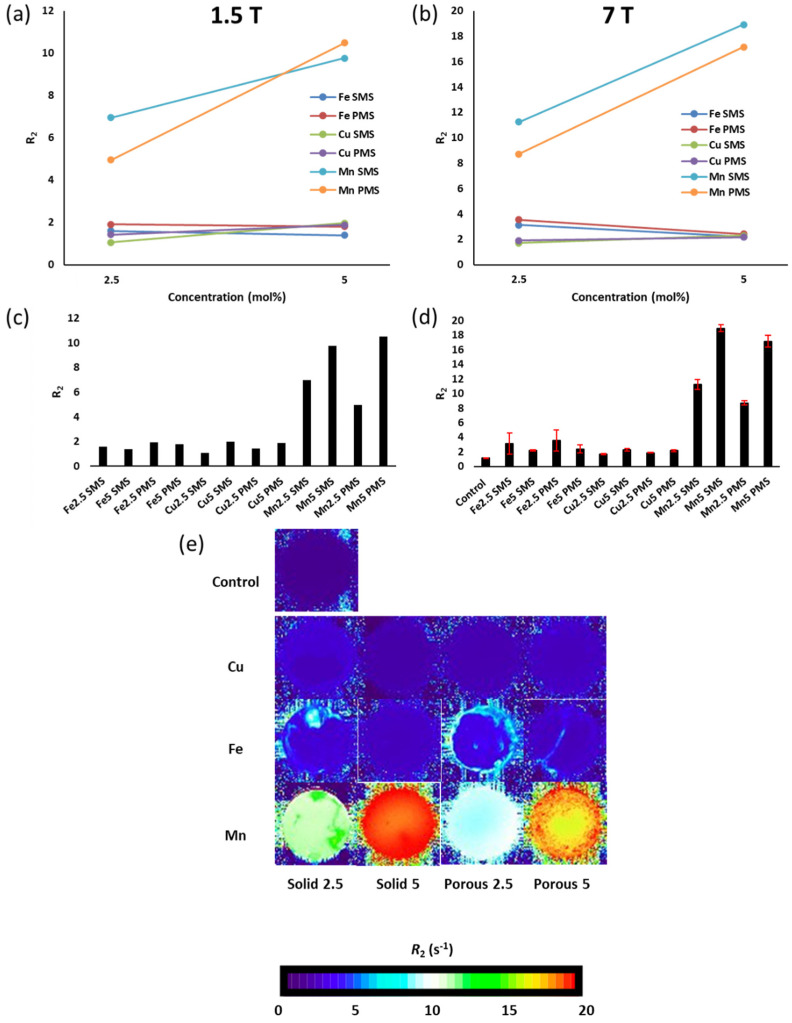
Transverse relaxivity (*R*_2_) measurements and associated MRI relaxation images showing hydroxymethyl cellulose suspension of solid microspheres (SMS) and porous microspheres (PMS) loaded with iron (Fe), copper (Cu), and manganese (Mn) assessed via 1.5 T (**a**,**c**) and 7 T (**b**,**d**,**e**) MRI systems. (**a**,**b**) highlight *R*_2_ measurements as a function of molar concentration; (**c**,**d**) *R*_2_ relaxation times for SMS and PMS. 7 T MRI measurements are compared with s hydroxymethyl cellulose solution as a way of control; and (**e**) 7 T MRI system *R*_2_ relaxation MRI mappings for SMS and PMS suspensions.

Figure 8 presents the transverse relaxation rate *(R*_2_) measurements and MRI relaxation maps corresponding to Fe, Cu, and Mn P45-based SMS and PMS samples, all individually suspended and evenly dispersed in a hydroxymethyl cellulose solution and assessed via 1.5 T and 7 T MRI systems. Figure 8a,b show the 1.5 T and 7 T transverse relaxation rates, respectively, for P45-SMS and P45-PMS as a function of Mn, Fe and Co molar concentrations (2.5 mol% and 5 mol%). Manganese 5 mol% samples show a clear increase in transverse relaxation time when compared to lower concentration Mn 2.5% mol. Moreover, Mn5 PMS’s *R*_2_ value surpassed that of the Mn5 SMS, as shown in 1.5T (Figure 8a). Nevertheless, this behaviour was not observed for 7 T. This effect may be due to variations in the water exchange rate between SMS and PMS (100–200 µm), with more exchange rate optimisation in the lower field for porous particles. Further studies would be required to understand these properties in more detail. As shown in Figure 8e, the highest *R*_2_ relaxation values (~17–19 s^−1^) correspond to Mn5 microspheres. The phantom image corresponding to Mn5 PMS (same case for Fe2.5 solid and porous) exhibits varying intensities attributed to inhomogeneous distribution due to microsphere suspension settling. Tubes were flipped to redistribute samples, with values taken at a consistent height and averaged to minimise variability. Surprisingly, iron-based samples showed a dissimilar effect to manganese-based microspheres. Fe microspheres with 5 mol% concentrations showed a slightly reduced *R*_2_ value when compared to 2.5 mol%, as both SMS and PMS relaxation times decreased as a function of molar concentration. This trend was consistent for iron microspheres in both the 1.5 T and 7 T MRI measurements (Figure 8a–d). These small changes may be associated with the inclusion of small bubbles or minimal differences in Fe compositional distribution. Nevertheless, these changes are statistically insignificant. Regarding the porosity effect, Fe PMS revealed increased *R*_2_ values when compared to Fe-SMS. Moreover, for the case of copper-based microspheres (Figure 8a–d), an increase in the transverse relaxation rate was observed as a function of Cu concentrations, showing consistency at both 1.5 T and 7 T. Interestingly, copper PMS revealed similar *R*_2_ values to those of copper SMS. However, the overall transverse relaxivity of the copper-based particles was the lowest of the three metal ions tested.

**Figure 9:**
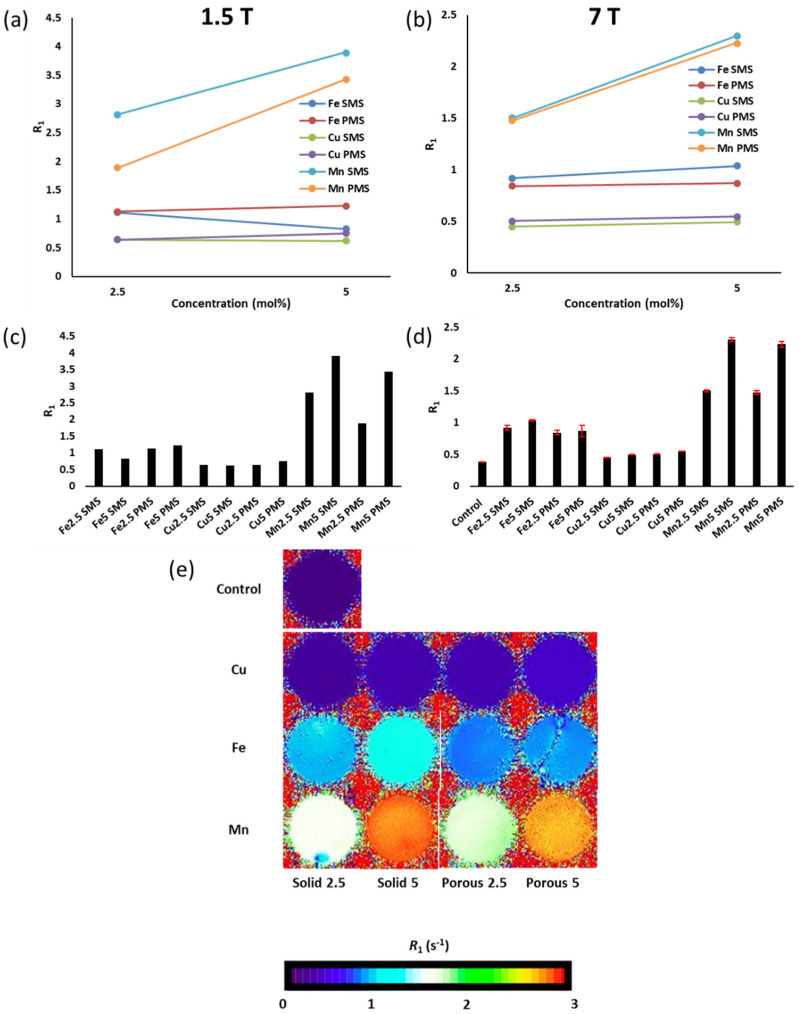
Longitudinal relaxivity (*R*_1_) measurements and associated MRI relaxation images showing hydroxymethyl cellulose suspensions of solid microspheres (SMS) and porous microspheres (PMS) loaded with iron (Fe), copper (Cu), and manganese (Mn) assessed via 1.5 T (**a**,**c**) and 7 T (**b**,**d**,**e**) MRI systems. (**a**,**b**) highlight *R*_1_ measurements as a function of molar concentration; (**c**,**d**) *R*_1_ relaxation times for SMS and PMS. 7 T measurements are compared with hydroxymethyl cellulose solution as way of control; and (**e**) 7 T MRI system *R*_1_ relaxation MRI images for SMS and PMS suspensions.

Figure 9 shows longitudinal relaxation (*R*_1_) measurements and MRI relaxation maps associated with Mn, Fe, and Cu P45-based SMS and PMS samples, again, all individually prepared in a hydroxymethyl cellulose solution and assessed via both 1.5T and 7T MRI systems. Measurements of solid and porous particles loaded with manganese revealed a significant *T*_1_ relaxivity when compared to the iron and copper-based microsphere suspensions (Figure 9a,b) and a control (Figure 9d). As shown in Figure 9e, the effect was pronounced for the Mn5 solid microspheres, which showed an *R*_1_ of 3.9 s^−1^ (1.5 T, Figure 9a) and 2.3 s^−1^ (7 T, Figure 9b), and for Mn5 porous microspheres with *R*_1_ values of 3.4 s^−1^ (1.5 T, Figure 9a) and 2.2 s^−1^ (7 T, Figure 9b). In the case of the iron-based SMS, the effect of concentration showed discrepancies between both MRI measurements (Figure 9a,b). Conversely, Fe PMS showed consistency in both 1.5 T and 7 T measurements, revealing a slight *R*_1_ increment increasing from 2.5 mol% to 5 mol%. For the case of copper, it was noted that porous microspheres showed increased levels of *R*_1_ when compared to solid counterparts. Nevertheless, *R*_1_ values were very close to those of the control (Figure 9d).

The Mn-based microspheres are the most promising contrast agents at both field strengths tested, with longitudinal (*r*_1_*)* and transverse (*r*_2_) relaxivities, including the relaxivity ratios (*r*_2_/*r*_1_), presented in Table 3. The porous and solid microspheres produced would perform reasonably well at both clinical and higher field strengths. The longitudinal relaxivity is lower in magnitude than clinical Gd contrast agents (*r*_1_~5 mM^−1^s^−1^) [31], though the varying nature of the proposed application and the likely large physiological differences between small molecules and microsphere materials make direct comparisons very challenging and somewhat misleading. More accurate determinations of the relaxivities would be generated with an increased range in the metal-doping quantities. The high *r*_2_/*r*_1_ ratio suggests that Mn microspheres would be suitable as transverse (*T*_2_) imaging agents at 7 T. Furthermore, this ratio is not high enough to preclude their use in longitudinal (*T*_1_) imaging using a 1.5 T system. The Fe and Cu doped microspheres, while displaying some relaxation enhancement, revealed relaxivities <0.3 mM^−1^s^−1^. However, the non-linear nature of the relaxation properties of the Fe/Cu systems with respect to concentration makes accurate calculations of relaxivities unfeasible and, as such, no such calculations are reported.

## 3. Discussion

### 3.1. Materials Properties

Glass-based microspheres (both dense and porous) have been manufactured via spray-drying [32], emulsification/solvent evaporation, and other methods [33]. Moreover, porous micro-scaffolds have been fabricated using additive manufacturing technologies [34,35]. However, such multiple-step methods are laborious and time-consuming, and present limited scalability [36] along with poor control over microsphere morphology, such as particle size and pore interconnectivity [37]. Alternatively, the flame spheroidisation process is a rapid, single-stage manufacturing process, suitable for the production of microspheres of controlled size, interconnected porosity, and compositional uniformity [38]. More recently, the flame spheroidisation process has also been used to develop ferromagnetic microspheres for hyperthermia applications [39]. Nevertheless, this is the first report on the production of PBG microspheres with modified compositions for MRI applications. 

The manufacture of solid microspheres was extremely rapid. SEM characterisation confirmed the transformation of irregularly shaped particles of phosphate glass into spherical morphologies. Amorphous structures were expected to appear in the prepared glass formulations post-processing into microspheres, and the expectation was confirmed by XRD analysis (see Figure 3). The starting phosphate glass formulations were doped with 2.5 and 5 mol% concentrations of the metal oxides Fe_2_O_3_, CuO, and MnO_2_, which were confirmed via EDX analysis to be within 1–2% of the targeted formulation value. Moreover, EDX mapping revealed a uniform elemental distribution among the SMS, highlighting the reproducibility of the flame spheroidisation manufacturing process employed.

The porous microspheres produced showed evidence of large surface pores and fully interconnected porosity among all the formulations investigated. It is suggested that the pore sizes obtained were similar to those of the porous phosphate glass microspheres reported in a previous study [37]. These microspheres showed an average pore size of 55 ± 8 µm, an interconnected porosity of 76 ± 5%, and surface pore areas ranging from 0.3 to 0.9 m^2^g^−1^. Nevertheless, the yield of porosity achieved revealed differences between the larger (150–200 µm) and smaller (100–150 µm) microsphere size ranges, with the larger range showing a higher pore quantity. It has been reported [40] that the formation of porous microspheres requires the entrapment and release of CO_2_ gas bubbles (obtained via decomposition of the carbonate porogen) from the molten glass particles exiting the flame spheroidisation process and cooling. Hence, an expansion mechanism is also associated with the formation of porous microspheres, driven by this CO_2_ gas entrapment/release as evidenced by the yield of the larger microsphere size range produced. 

Furthermore, the use of combined porogens for the development of porous microspheres accounted for the structural and compositional differences observed when compared to the starting glass and the solid microspheres produced. Notably, CaCO_3_ XRD peaks correlated well with the EDX elemental mappings, noting the presence of calcium-rich irregularly shaped particles containing an excess of unreacted CaCO_3_ (see Figure 6). However, no evidence of Na_2_CO_3_ was obtained via XRD analysis, suggesting consumption of the decomposed porogen (i.e., Na_2_O) had occurred, as indicated by compositional EDX data, which revealed higher levels of Na_2_O (Table 1) within the end products as compared to the starting glass formulation values (Table 4).

The NMR investigations revealed that the solid (dense) P45 phosphate glass microspheres mainly contained Q^2^ (metaphosphate) and Q^1^ (pyrophosphate) species. Both Q^2^ and Q^1^ provide controlled and slow degradation rates, especially when compared to phosphate rich Q^3^ ultraphosphate structures, which can degrade rapidly [41]. Similar profiles have previously been reported for PBGs with fixed 45mol% P_2_O_5_, revealing an 80:20 ratio of Q_2_:Q_1_ species [42]. In the present study, the NMR data revealed different Q^2^:Q^1^ ratios. Among the oxides studied, Fe showed the most pronounced effect. From 2.5 mol% (55:45 of Q^2^:Q^1^) to 5 mol% (39:61 of Q^2^:Q^1^), the Q^2^ species were observed to have progressively decreased while Q^1^ species increased, suggesting a shortening of phosphate chain lengths. In the case of Cu, the effect was minimal. From 2.5 mol% (68:32 of Q^2^:Q^1^) to 5 mol% (67:33 of Q^2^:Q^1^), the Q^2^:Q^1^ ratios remained very similar. For the case of Mn-based microspheres, although they showed Q^0^ (orthophosphates) species for the 2.5 mol% samples, these were dominated by Q^1^ species. Notably, the species ratios found in our study were considerably different from those observed in a previous study [42]. These changes were attributed to the presence of modifier oxides in the glasses, which promoted the formation of more non-bridging oxygen atoms to balance the positive charge [43] induced by Fe^3+^, Cu^2+^, and Mn^4+^ cations.

In comparison to the solid microspheres, the NMR studies of porous P45 phosphate glass microspheres revealed the presence of Q^0^ orthophosphate species and a subsequent decrease of Q^2^ species (see Table 2). These profiles were also indicative of network depolymerisation, which occurred to achieve a higher proportion of non-bridging oxygen atoms [43]. Again, it was suggested that the increased quantity of non-bridging oxygen atoms emerged in response to the unbalanced positive charge induced by the incorporation of additional calcium and sodium cations (arising from the CaCO_3_ and Na_2_CO_3_ porogens) into the glass structure [44]. Importantly, orthophosphates exhibit more controlled degradation rates than Q^3^ species [45] and are also essential for bone remodelling processes [41].

Furthermore, the degradation profiles obtained (see Figure 7) indicated no significant variations in mass losses between the SMS and PMS samples. Nevertheless, it was expected that the PMS would exhibit greater degradation than SMS, due to their increased surface area. However, our studies showed a mass decrease (~8%) for the SMS and a (~1%) decrease for the PMS by day 28. As such, a follow-up particle size-based degradation study was conducted, which revealed a slight size reduction over time for both SMS and PMS with no statistically significant (*p* > 0.05) differences between them. As such, we hypothesise that SMS may have experienced precipitation build-up during the mass loss study, potentially influencing the mass loss observed. There was also evidence for the presence of some porogen remnants remaining in the PMS from XRD analysis (although an acid wash protocol was used). The porogen remnants were noted to have affected the pH of the immersion media, which could also have influenced the outcomes observed.

### 3.2. MRI Analysis

This first study on flame spheroidisation of fully resorbable phosphate-based glasses doped with copper, iron and manganese oxides for MRI applications revealed that compositional variations of the microspheres produced (as noted above), along with physical properties such as particle size and porosity, can influence the *R*_1_ and *R*_2_ relaxation rates. The MR imaging studies demonstrated the potential for phosphate glass microspheres to act as MR contrast agents. While the Cu and Fe particles displayed limited MR responses, the Mn particles showed promising relaxivity at field strengths that are both clinically relevant (1.5 T) and common in preclinical research (7 T). These results are consistent with previous work using Mn and Cu salts in solution, in which Mn showed an *R*_1_ relaxivity one order of magnitude larger and an *R*_2_ relaxivity two orders of magnitude larger than the corresponding relaxivities of Cu [46]. The Mn-based particles showed a concentration-dependent increase in both longitudinal and transverse relaxation rates. The results indicated that there were sufficient paramagnetic ions in the Mn microspheres to significantly enhance relaxation in the surrounding bulk water. There were also subtle field-dependent differences observed between the microspheres. The relatively similar increase in both longitudinal and transverse relaxation appeared to be more in line with small molecule *T*_1_ agents, rather than superparamagnetic iron oxide nanoparticles. As with small molecule Gd/Mn complexes, the effect on relaxation is likely a complex interplay between surface water interactions (inner/outer/second sphere), water exchange (*τ*_m_), and the rotational correlation time (*τ*_r_) of the microspheres [47]. It is likely that these contributions varied between the porous and solid particles, with the porous microspheres having a limited effect on *R*_1_ and *R*_2_. This could be attributed to the rapid increase in the viscosity of the hydroxymethyl cellulose/PMS suspension within 2 min, which slows water exchange. Although water can penetrate the pores, it does not exchange rapidly enough with the bulk water to significantly enhance relaxation. Further focused studies will be required to deconvolute this aspect more thoroughly. Overall, the Mn-based particles showed much promise for future applications in contrast-enhanced imaging studies. In addition, these resorbable materials are regarded as much safer and more environmentally friendly materials than Gd agents [48].

The selection of oxide precursors for this investigation relates to their biocompatibility and/or magnetic properties and their potential suitability for MRI applications [49]. In the case of iron, an abundant element in the human body (e.g., present in haemoglobin), the appropriate doses of this element are facile to metabolise [50] and possess attractive magnetic properties, such as elevated magnetic saturation. However, studies show that pure iron-based microspheres displayed a limited MR response (as highlighted above). Furthermore, studies have shown that adding iron oxides to PBGs can result in Fe_2_O_3_, hence the low MRI relaxivities observed [51]. Alternatively, PBGs with dispersions of Fe_3_O_4_ ferromagnetic domains have been developed [39], but have not yet been explored for MRI applications. Although a magnetometry analysis was not carried out for the PBGs used in this study, a previous study [39] from our group investigating the magnetometry of PBG alone vs PBG-Fe_3_O_4_ revealed typical hysteresis loops indicative of ferromagnetic behaviour (saturation magnetisation 4 Am^2^ kg^−1^; coercive field 5.8 kA m^−1^). Copper (Cu) oxides have remained mostly unexplored as MRI CAs. However, copper is a vital nutrient for humans, serving in several biological processes such as iron absorption, bone formation, and brain function [52,53]. Copper also offers paramagnetic expression, which could be attractive for MRI applications [54]. Indeed, unpaired electrons (including those of copper ions) can create a strong relaxivity effect [55]. Manganese (Mn^2+^) based agents are one of the more widely researched paramagnetic *T*_1_ contrast agents for MRI, mainly due to their effectiveness in enhancing *R*_1_ signals and reducing toxicity (as compared to gadolinium agents) [56]. For example, MnCl_2_ contrast agents have been used successfully in vivo in combination with a manganese-enhanced MRI (MEMRI) system. A study by Yang et al. [57] reported that lower doses of Mn^2+^ from a slow-release agent could mitigate toxicity issues. Therefore, embedding a network modifier oxide (e.g., MnO_2_) within a bioactive, resorbable matrix (such as P45 phosphate-based glasses) would offer significantly reduced toxicity issues as the formulations could easily be tailored to regulate the ion release rate. Indeed, MnO_2_ (bio)glasses have previously been produced and demonstrated cytocompatibility [58,59] and biocompatibility in in vivo studies [60]. In particular, a phosphate glass matrix with low amounts of modifying oxides, including MnO_2_, showed ion release control that extended over several months depending on the composition and targeted applications [61]. It is also worth noting that for GI applications, the toxicity concerns are reduced even further as the materials would be expected to pass rapidly through the GI system, being excreted through the natural pathways of the body. In particular, Mn has been explored as a contrast agent for the detection of anatomical structures, such as in the hepatic and cardiac areas [62]. Additionally, phosphate glasses coupled with Fe or Cu oxides have been investigated for healthcare applications. For example, the biocompatibility of iron phosphate-based glasses has been demonstrated [51], and phosphate-based glasses doped with CuO have showed relevance in wound healing applications [63]. The in vitro activity and cytocompatibility of Mn incorporated within bioactive phosphate-based glass has also shown promising results for bone tissue regeneration [64].

**Figure 10:**
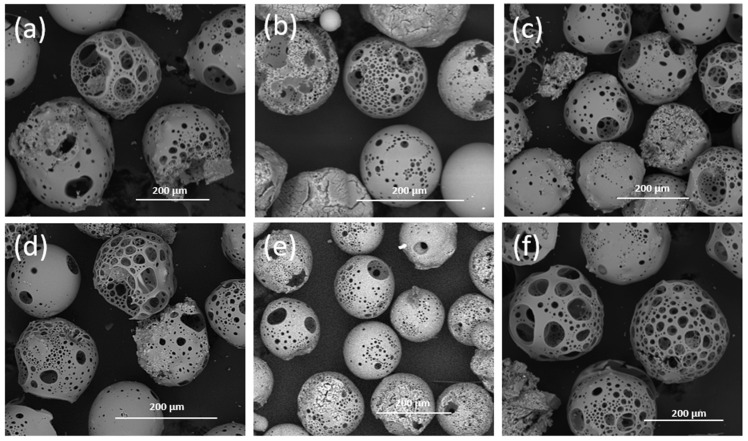
High-magnification SEM images of flame spheroidised-processed porous microspheres (PMS), in the range of 150–200 µm, with modified compositions: Fe–(**a**) 2.5%, (**d**) 5%; Cu–(**b**) 2.5%, (**e**) 5%; and Mn–(**c**) 2.5%, (**f**) 5%, extracted from the sample sets presented in Figure 2.

It is also noted that the PMS were promising candidates for MRI theranostic applications. Figure 10 shows SEM images of the porous PBG microspheres in detail, extracted from the sample sets presented in Figure 1. Their porosity enables the incorporation of payloads (such as drugs, proteins, biologics, etc.). This opens up the opportunity for porous microspheres to act as release agents in a controlled manner e.g., via controlled degradation rates. This controlled release could allow for the targeted and controlled delivery of pharmaceutics/therapeutics to the GI tract. For example, PMS could be explored for treating inflammatory bowel disease [65] and vascular structures and anomalies within the GI tract [66] by releasing incorporated drugs as they degrade. SMS could be explored for MRI-guided bariatric artery embolisation [67]. Remarkably, bioactive glasses have also shown wound healing capabilities when administered to GI mucosa tissue [68]. Furthermore, their magnetic relaxation profiles could enable the simultaneous monitoring of treatment responses in real-time alongside soft tissue repair/regeneration. In this context, it is suggested that the porous PBG microspheres developed in this study open up further opportunities for controlled drug release within GI and other target tissue applications.

## 4. Experimental

### 4.1. Materials

Resorbable phosphate-based glass (PBG) formulations were prepared using the following precursors: NaH_2_PO_4_ (S5011), CaHPO_4_ (C7263), and P_2_O_5_ (214701) (Merck), including additional oxides (i.e., Fe_2_O_3_ (310050), CuO (241741), and MnO_2_ (217646)) (Merck) as starting materials. 

### 4.2. Glass Formulation

Specific amounts of each precursor (according to the formulations produced as highlighted in Table 4) were placed into a 100 mL volume Pt/5%Au crucible. The crucible was then placed into a furnace at 350 °C for 30 min (for drying) prior to melting in another furnace at 1150 °C for 90 min. The molten glass was then poured onto a metal plate and allowed to cool to room temperature.

**Table 4:** Expected formulations (mol%) for melt-quenched phosphate glasses (P45) along with their respective glass codes used in this study.

Glass Code	P_2_O_5_/mol%	CaO/mol%	Na_2_O/mol%	Fe_2_O_3_/mol%	CuO/mol%	MnO_2_/mol%
P45-Fe2.5	45	40	12.5	2.5	-	-
P45-Fe5	45	40	10	5	-	-
P45-Cu2.5	45	40	12.5	-	2.5	-
P45-Cu5	45	40	10	-	5	-
P45-Mn2.5	45	40	12.5	-	-	2.5
P45-Mn5	45	40	10	-	-	5

### 4.3. Microsphere Manufacture 

After cooling, the prepared PBGs were ground into microparticles using a ball milling machine (Retsch PM100 Ball Mill, Retsch GmbH, Haan, Germany) and sieved to a range of between 63–125 µm (VWR International, Radnor, PA, USA). Solid (i.e., bulk, dense) microspheres (SMS) were produced via a flame spheroidisation process as previously reported [40]. Glass particles were briefly fed into an oxygen/acetylene flame (thermal spray gun, MK 74, Metallisation Ltd., West Midlands, UK). Porous glass microspheres (PMS) were produced by combining ground up glass particles with CaCO_3_ and Na_2_CO_3_ (mass ratio 1:3) using a vortex mixer. The prepared powders were then mixed with phosphate-based glass microparticles (63–125 µm) (porogen powders/glass powder 3:1 mass ratio) and processed through the flame spheroidisation method as detailed above. After spheroidisation, the microspheres were subjected to a wash-step using acetic acid (2 M) for 2 min with gentle stirring, and then dried in an oven at 50 °C for 24 h.

### 4.4. Sample Preparation for EDX Analysis

Cross-sectional analyses of the microspheres were performed by embedding the microspheres in a cold set of epoxy resin and polished with SiC paper, followed by further polishing with diamond cloth. Industrial methylated spirit (IMS) was used as an eluent during polishing. The samples were then dried before coating. A platinum coating was applied using a polaron sputter coater (SC7640, Lewes, UK) to prevent image distortion due to charging.

### 4.5. Microsphere Characterisation

Scanning electron microscopy (SEM, Phillips FEI XL30, Hillsboro, OR, USA) was conducted using an accelerating voltage of 15 KV and a working distance of 10 mm to examine the material’s morphology and confirm particle sizes before and after processing.

XRD analysis was performed in order to investigate the amorphous nature of the microspheres produced. The samples were analysed using a Bruker AXS–D8 Advance powder diffractometer (Coventry, UK) in flat plate geometry using Ni-filtered Cu-Kα radiation (λ = 0.15418 nm), operated at 40 kV and 35 mA. Data were collected at 2θ values from 10° to 70°, with a step size of 0.1° and a count time of 5 s. The phases were identified using the EVA software (DIFFRACplus Suite, Bruker AXS) (https://www.bruker.com/en/products-and-solutions/diffractometers-and-x-ray-microscopes/x-ray-diffractometers/diffrac-suite-software/diffrac-eva.html) and the International Centre for Diffraction Database (2005).

Quantitative EDX analysis of the samples was performed using an Oxford Instruments INCA EDX system with a Si–Li crystal detector. The EDX spectrometer was attached to the Philips XL30 scanning electron microscope (FEI Company, Hillsboro, OR, USA) and was operated in secondary electron imaging mode with an accelerating voltage of 10 kV, working distance of 10 mm, and system resolution of 60 eV. 

The ^31^P NMR spectra were recorded using a Bruker Advance III 600 MHz spectrometer (Coventry, UK) equipped with a triple resonance 2.5 mm MAS probe spinning at 30.0 kHz at room temperature. The ^31p^π2 pulse duration was 3.0 µs, the spectral width was 150 kHz, and the acquisition time was 27.3 ms. Relaxation times were measured using a standard saturation recovery sequence, consisting of a saturation block of multiple 90° pulses followed by an increasing recovery time and a final 90° and acquisition.

### 4.6. Degradation Studies

Degradation studies of both SMS and PMS were conducted based on mass loss, size, and pH changes of the media over a 28 day period. 

Mass-loss measurements were performed using 400 mg of microspheres (100–200 µm) for each formulation, placed inside a glass vial with milli-Q water (40 mL) as the medium. The vials were placed in an orbital shaker incubator (37 °C) and agitated (120 rpm). The mass of each formulation was measured before and after degradation at each time point (1, 3, 7, 14, 21, and 28 days). The microspheres were filtered and dried in an oven (50 °C; 24 h) before weight measurement. 

For the methodology employed to analyse size changes, both the SMS and PMS were selected from a very narrow size range (63–71 µm) within each formulation, placed in glass vials filled with milli-Q water, and incubated in an orbital shaker (37 °C; 120 rpm). At each time point (day 1, 3, 7, 14, 21, and 28) the microspheres were filtered and dried in an oven (50 °C; 24 h). The dried microspheres were then imaged and measured using electron microscopy (SEM, Phillips FEI XL30, Hillsboro, OR, USA). The particle size distribution was analysed via ImageJ 1.51h software (National Institutes of Health, Bethesda, MD, USA) [69,70].

The pH of the medium was also measured using a pH meter (Mettler Toledo, Greifensee, Switzerland). Here the medium was refreshed at each time point, with three replicate measurements taken at each time point.

### 4.7. Magnetic Resonance Imaging

#### 4.7.1. Suspension Preparation

For MRI analysis, the microspheres needed to be suspended uniformly to prevent agglomeration. Hydroxymethyl cellulose was selected as the suspension medium due to its transparency and the controllability of its viscosity. Samples of each of the microsphere formulations produced were individually suspended in a 3.1% *w*/*w* hydroxyethyl cellulose water/sodium chloride solution at 2.5% *w*/*w* and 5% *w*/*w* microsphere concentrations. A stock solution was prepared by dissolving 330 mg of sodium chloride powder in 40 mL of water using a laboratory magnet stirrer. Then 1.2 g of hydroxyethyl cellulose powder was gradually incorporated and stirred for 10 min. Subsequently, 240 mg of microspheres (100–200 µm) were steadily incorporated into the suspension and stirred for 2 min. This formed a moderately viscous gel to aid the even and uniform suspension of the microspheres. The microsphere solution was then placed in 50 mL conical centrifugal lab tubes (Falcon tubes) for testing in a 1.5 T and 7 T MRI scanner. 

The Falcon tubes were placed in a MultiSample 190F phantom holder (Gold Standard Phantoms, Sheffield, UK), which is cylindrical in construction and flooded with a solution of nickel chloride and sodium chloride in water to minimise susceptibility effects.

#### 4.7.2. MRI Analysis

The phantom holder was placed in an 8-element parallel imaging head coil and imaged at room temperature on a 1.5 T MRI scanner (GE Healthcare, Milwaukee, WI, USA). The longitudinal relaxation time *T*_1_ was measured using an inversion recovery with a spin echo (SE) echo planar imaging (EPI) readout. Twenty-three inversion times (TI) were used, ranging between 50 and 4000 ms. The sequence used a 5 mm slice thickness, a 128 × 128 acquisition matrix with an in-plane acquired resolution of 2.3 mm × 2.3 mm, a repetition time (TR)/echo time (TE) of 5000 ms/18.4 ms, and a flip angle of 90°. The transverse relaxation time *T*_2_ was measured using a SE EPI sequence using 18 TEs ranging between 20 and 1000 ms. The *T*_2_ measurement sequence used a 5 mm slice thickness, a 128 × 128 acquisition matrix with an in-plane acquired resolution of 2.3 mm × 2.3 mm, a TR of 1200 ms, and a flip angle of 90°. *T*_1_ and *T*_2_ were calculated using a two-parameter non-linear least squares fit of the longitudinal signal recovery and transverse signal decay, respectively, to the average signal from a region of interest for each sample tube. 

The same samples were also measured in a 7 T scanner, using a 30 cm bore Bruker Biospec 70/30 MR scanner with a Bruker Avance III Console (Bruker BioSpin, Ettlingen, Germany). A 2 mm slice was imaged in both sagittal and coronal orientations through the centre of the tubes with data matrices of 256 × 128 and a field of view of 10 × 5 cm^2^. The *T*_1_ and *T*_2_ relaxation times were calculated using a RARE VTR sequence (flip angle = 180°) with six TRs between 225 and 10,000 ms and eight TEs ranging from 6.8 to 103 ms. Fiji/Image J was used to reconstruct the images, and the relaxation times were calculated by fitting the image intensity data to the exponential decay curves using the MRI Analysis Calculator plugin (Karl Schmidt).

As the 7T scanner is significantly smaller than the clinical 1.5T system used, the use of the phantom holder was not feasible due to machine size limitations. Therefore, each Falcon tube was imaged individually, including the control sample. Importantly, the phantom can hold only 12 samples (6 solid microsphere samples and 6 porous microsphere samples). For instance, a control sample was included only in the 7 T dataset.

Relaxation times (*T*_1_/*T*_2_) were converted to relaxation rates (*R*_1_/*R*_2_) for presentation, where *R*_1_ = 1/*T*_1_ and *R*_2_ = 1/*T*_2_, because relaxation rates are linearly related to microsphere concentration [71].

## 5. Conclusions

Dense (i.e., solid) and highly porous phosphate-based microspheres were developed from melt-quenched P45 glass formulations doped with Mn, Cu, and Fe oxides via a single-stage flame spheroidisation process. Complementary SEM, XRD, and EDX investigations confirmed the development of dense (63–200 µm) and porous (100–200 µm) P45-based microspheres, with evenly distributed concentrations of Fe_2_O_3_, CuO, and MnO_2_. The microspheres compositions showed consistency with prepared glass formulations (2.5 and 5 mol%). Moreover, NMR investigations confirmed the development of Q^2^ (metaphosphate) and Q^1^ (pyrophosphate) species for SMS, and Q^1^ and Q^0^ (orthophosphate) species for PMS, all associated with controlled and regulated degradation rates. The 28-day degradation, pH, and mass-loss studies showed no significant differences among the microspheres (63–71 µm) tested. Among the microsphere compositions investigated, 5mol% doped manganese PBGs showed clinical (7 T) and preclinical (1.5 T) relevance in MRI measurements, with enhanced levels of *R*_2_ (9.7–10.5 s^−1^ for 1.5 T; 17.1–18.9 s^−1^ for 7 T) and *R*_1_ (3.4–3.9 s^−1^ for 1.5 T; 2.2–2.3 s^−1^ for 7 T) when compared to iron and copper-based microsphere relaxations. It is suggested that the development of bioactive phosphate-based microspheres with modified compositions opens up novel opportunities for the manufacture of resorbable MRI contrast-enhancement oral-delivery products, with tuneable relaxivity properties and theranostic capabilities to deliver payloads (e.g., drugs/other biologics) by exploiting the porous microspheres produced.

## Figures and Tables

**Table 1:** EDX molar concentrations (weight %) of flame spheroidised solid and porous glass microspheres P45, with modified Fe, Cu, and Mn compositions.

Sample		P_2_O_5/_wt%	CaO/wt%	Na_2_O/wt%	Fe_2_O_3_/wt%	CuO/wt%	MnO_2_/wt%
SMS	P45-Fe2.5	41 ± 4	44 ± 4	13 ± 1	2	-	-
	P45-Fe5	33 ± 1	51 ± 1	11	5	-	-

	P45-Cu2.5	30 ± 4	60 ± 7	7 ± 3	-	3	-
	P45-Cu5	29 ± 3	57 ± 5	8 ± 3	-	6	-

	P45-Mn2.5	34 ± 1	50 ± 1	12	-	-	3
	P45-Mn5	34 ± 2	51 ± 2	9 ± 1	-	-	6

PMS	P45-Fe2.5	35 ± 3	45 ± 4	17 ± 3	3	-	-
	P45-Fe5	35 ± 3	46 ± 6	14 ± 8	6 ± 1	-	-

	P45-Cu2.5	37 ± 2	39 ± 4	21 ± 5	-	2	-
	P45-Cu5	37 ± 4	41 ± 6	17 ± 5	-	5 ± 3	-

	P45-Mn2.5	36 ± 4	49 ± 5	12 ± 2	-	-	2
	P45-Mn5	38 ± 2	44 ± 2	13 ± 3	-	-	5

**Table 2:** NMR peak positions and relative proportions (%) of Q^0^, Q^1^, and Q^2^ species in both solid and porous glass microspheres P45 with Fe, Cu, and Mn.

Samples	SMS			PMS		
	Q^0^	Q^1^	Q^2^	Q^0^	Q^1^	Q^2^
P45-Fe2.5 Shift/ppm X^n^/%	- -	−8.1 44.8	−22.8 55.2	3.3 8.7	−6.2 50.1	−22.3 40.6
P45-Fe5 Shift/ppm X^n^/%	- -	−8.5 61.4	−20.9 38.6	3.8 24.4	−5.3 37.5	−20.8 38.1
P45-Cu2.5 Shift/ppm X^n^/%	- -	−8.0 31.9	−23.9 68.1	2.8 11.1	−6.6 34.4	−23.1 54.3
P45-Cu5 Shift/ppm X^n^/%	- -	−7.9 32.6	−23.8 67.4	3.9 2.2	−6.5 32.7	−22.8 65.1
P45-Mn2.5 Shift/ppm X^n^/%	−9 -	−22 -	- -	3 -	−7 -	−21 -
P45-Mn5 Shift/ppm X^n^/%	- -	−22 -	- -	2.5 -	−8 -	−22 -

**Table 3:** Longitudinal (*r*_1_) and transverse (*r*_2_) relaxivities for Mn-based microspheres at both 1.5 and 7 T, including *r*_2_/*r*_1_ ratios. Values calculated from the change in relaxation against increasing concentration of Mn, according to the equation *R*_1/2_ = *r*_1/2_ * [Mn]. (Relaxivity values are based on the total Mn concentration in the sample).

Samples	*r*_1_ (mM^−1^s^−1^)		*r*_2_ (mM^−1^s^−1^)		*r*_2/_ *r*_1_	
	1.5 T	7 T	1.5 T	7 T	1.5 T	7 T
Mn SMS	0.5	0.5	2.1	4.3	4.1	8.6
Mn PMS	0.9	0.5	2.7	4.6	3.0	9.2

## Data Availability

The raw data supporting the results in the present study are available from the corresponding author upon reasonable request.

## Supplementary Materials

The following supporting information can be downloaded at: https://www.mdpi.com/article/10.3390/molecules29184296/s1, Figure S1: ^31^P Nuclear magnetic resonance (NMR) spectroscopy for solid microspheres (SMS) and porous microspheres (PMS) with iron; Figure S2: ^31^P Nuclear magnetic resonance (NMR) spectroscopy for solid microspheres (SMS) and porous microspheres (PMS) with copper; Figure S3: ^31^P Nuclear magnetic resonance (NMR) spectroscopy for solid microspheres (SMS) and porous microspheres (PMS) with manganese.
