# Supplementary material for: Development of Resorbable Phosphate-Based Glass Microspheres as MRI Contrast Media Agents
Source: Molecules. 2024 Sep 10;29(18):4296. doi: 10.3390/molecules29184296 (PMC11434598; doi:10.3390/molecules29184296)
Supplement: Supplementary file 1 [file molecules-29-04296-s001.zip › molecules-3168785-supplementary.pdf]

Electronic Supplementary Information

Development of Resorbable Phosphate-Based Glass Microspheres as MRI Contrast Media Agents

Jesús Molinar-Díaz[1,2], Andi Arjuna[1,3], Nichola Abrehart[4], Alison McLellan[5], Roy Harris[6], Md Towhidul Islam[1], Ahlam Alzaidi[7], Chris R. Bradley[8], Charlotte Gidman[5], Malcolm J. W. Prior[8], Jeremy Titman[5], Nicholas P. Blockley[7], Peter Harvey[5,8], Luca Marciani[4] and Ifty Ahmed[1*]

[1] Advanced Materials Research Group, Faculty of Engineering, University of Nottingham, NG7 2RD, UK; jesus.molinardiaz3@nottingham.ac.uk (J.M.-D.), andi.arjuna@nottingham.ac.uk (A.A.), towhid.islam@nottingham.ac.uk (M.T.I.)
[2] Composites Research Group, Faculty of Engineering, University of Nottingham, NG7 2GX, UK
[3] Faculty of Pharmacy, Hasanuddin University, Makassar, 90245, Indonesia
[4] Nottingham Digestive Diseases Centre, Translational Medical Sciences, School of Medicine, University of Nottingham NG7 2UH, UK; nichola.abrehart@nottingham.ac.uk (N.A.), luca.marciani@nottingham.ac.uk (L.M.)
[5] School of Chemistry, University of Nottingham, Nottingham NG7 2RD, UK; alisondalymclellan@live.com (A.M.), charlotte.gidman1@nottingham.ac.uk (C.G.), jeremy.titman@nottingham.ac.uk (J.T.), peter.harvey@nottingham.ac.uk (P.H.)
[6] Research Design Service East Midlands, Queen's Medical Centre, Nottingham NG7 2UH, UK; roy.harris1@nottingham.ac.uk (R.H.)
[7] School of Life Sciences, University of Nottingham Medical School, Queen's Medical Centre, Nottingham NG7 2UH, UK; lpxaaa@nottingham.ac.uk (A.A.), nicholas.blockley@nottingham.ac.uk (N.B.)
[8] Sir Peter Mansfield Imaging Centre, School of Medicine, University of Nottingham NG7 2QX, UK; christopher.bradley@nottingham.ac.uk (C.R.B.), malcolm.prior@nottingham.ac.uk (M.J.W.P.)
[*] Correspondence: ifty.ahmed@nottingham.ac.uk (I.A.)
Corresponding author. E-mail address: ifty.ahmed@nottingham.ac.uk

Keywords: Magnetic Resonance Imaging, phosphate-based glasses, oral contrast agents, porous microspheres, resorbable materials.

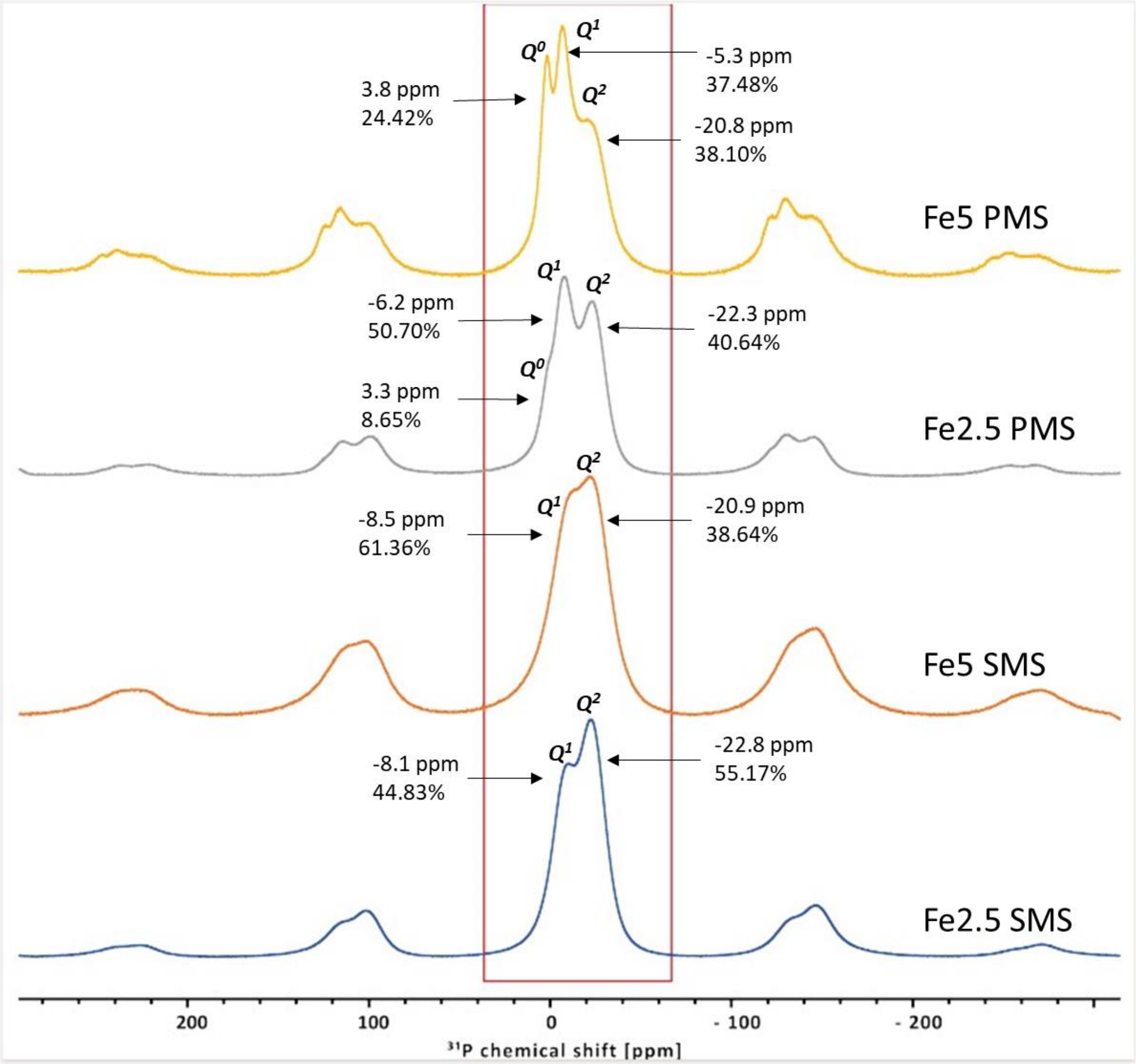

Figure S1. *^{31}P Nuclear magnetic resonance (NMR) spectroscopy for solid microspheres (SMS) and porous microspheres (PMS) with **iron**.*

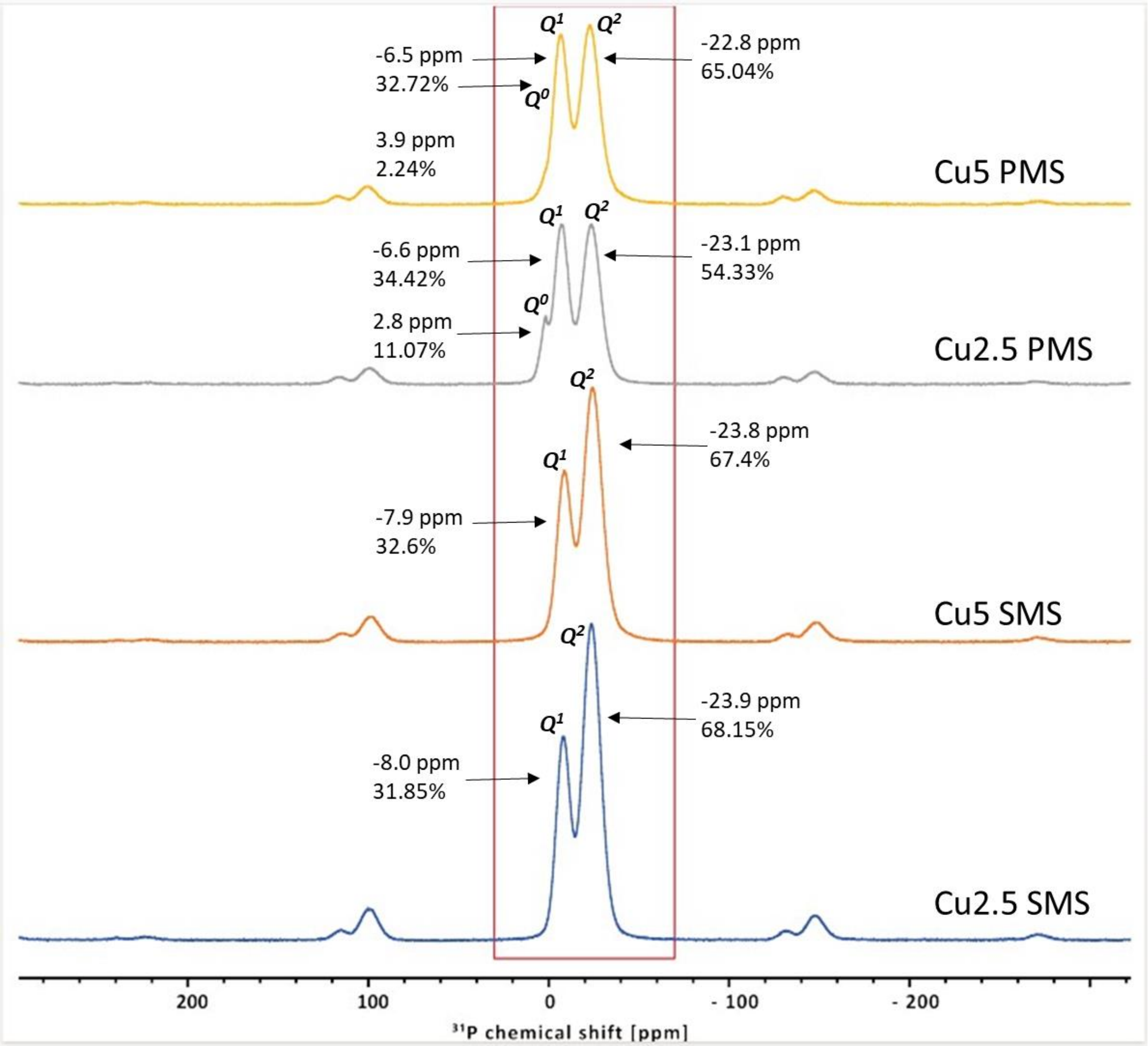

Figure S2. *^{31}P Nuclear magnetic resonance (NMR) spectroscopy for solid microspheres (SMS) and porous microspheres (PMS) with **copper**.*

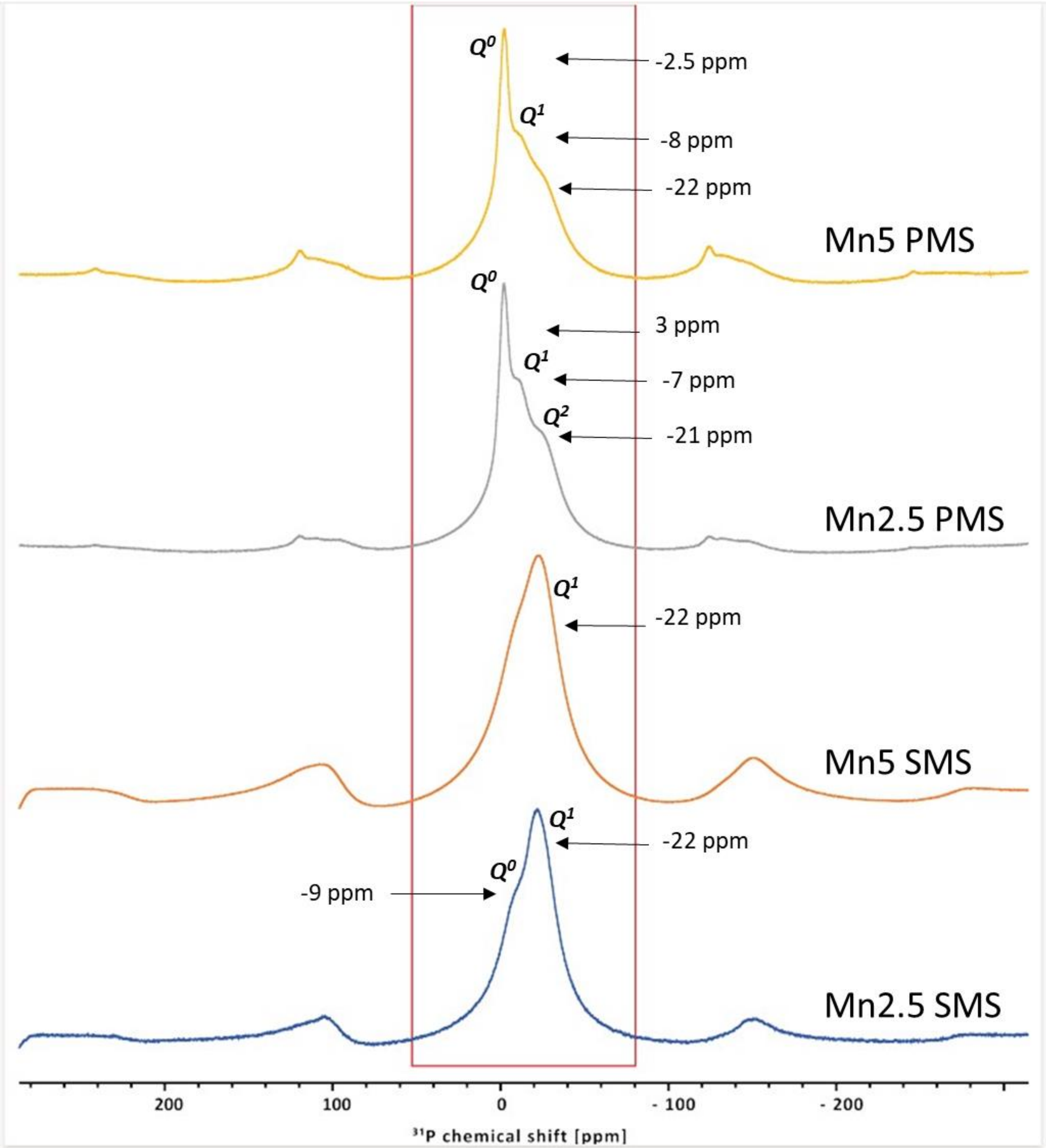

Figure S3. *^{31}P Nuclear magnetic resonance (NMR) spectroscopy for solid microspheres (SMS) and porous microspheres (PMS) with* ***manganese****.*
